# Effect of probiotics, prebiotics and synbiotics for chronic bronchitis or chronic obstructive pulmonary disease: A protocol for systematic review and meta-analysis

**DOI:** 10.1097/MD.0000000000023045

**Published:** 2020-11-06

**Authors:** Caixia Pei, Yongcan Wu, Xiaomin Wang, Fei Wang, Liyun Liu

**Affiliations:** a Department of Geriatrics; b Department of Respiratory Medicine, Hospital of Chengdu University of Traditional Chinese Medicine, No. 39 Shi-er-qiao Road; c General Practitioners’ Training Center of Sichuan Province, No. 19, Lower Wangjiaguai St., Chengdu, Sichuan Province, People's Republic of China.

**Keywords:** chronic bronchitis, chronic obstructive pulmonary disease, prebiotics, probiotics, synbiotics

## Abstract

**Background:**

Probiotics, prebiotics and synbiotics have been widely used in the treatment of respiratory diseases, but their clinical efficacy for treating chronic bronchitis (CB) or chronic obstructive pulmonary disease (COPD) has not been well studied.

**Methods:**

The following electronic databases will be searched for eligible randomized controlled trials: the Cochrane Library, EMBASE, MEDLINE, PubMed, Scopus, the Web of Science, the China National Knowledge Infrastructure, the Wanfang database, and the China Science and Technology Journal database (VIP Information Network). We will search these electronic databases weekly and extract relevant data from their inception dates until September 30, 2020. Risk of publication bias will be evaluated by the Cochrane Handbook for Systematic Reviews of Interventions. Data synthesis will be conducted using Review Manager (RevMan) version 5.3 software. Sensitivity and quality of evidence analyses will be conducted.

**Results:**

This systematic review and meta-analysis will provide a high-quality synthesis from existing evidence for estimating the efficacy and safety of probiotics, prebiotics and synbiotics in the treatment of CB or COPD.

**Conclusion:**

This systematic review and meta-analysis will provide reliable and accurate evidence to guide the use of probiotics, prebiotics and synbiotics in the treatment of CB or COPD.

**Registration OSF registration number:**

DOI 10.17605/OSF.IO/SP35M.

## 1 Introduction

Chronic bronchitis (CB) is medically defined as chronic cough and sputum production for 3 months every year for 2 consecutive years.^[1]^ Chronic obstructive pulmonary disease (COPD) is a major public health problem, typically characterized by persistent airflow limitation and increased airway inflammation, which includes pulmonary diseases such as CB and bronchial asthma. The prevalence of COPD has risen steadily over the past several decades. COPD has become the third leading cause of disease death worldwide with a significant economic burden.^[1,2]^ Smoking has been identified as the most significant risk factor for development of CB^[3]^; however, other potential risk factors include air pollution, biomass fuels, dusts and chemical fumes.^[4,5]^ Among individuals who reported smoking continuously over several decades, the incidence of CB was 42%.^[6]^

Chronic lung diseases, such as asthma and COPD, often co-occur with chronic gastrointestinal tract diseases, such as inflammatory bowel disease (IBD) or irritable bowel syndrome.^[7,8]^ In addition, studies have shown that up to 50% of adult IBD patients have pulmonary involvement, such as inflammation or lung function damage.^[9,10]^ These disease interrelationships suggest that there is an important bidirectional communication between the lung and the gut, known as the gut-lung axis, which may be influenced in part by communities of microbiota. The human microbiome is believed to contribute to homeostasis and disease, especially in the gut. Moreover, evidence has shown that the gut microbiota vary with smoking status.^[11]^ Recently, a growing body of evidence has demonstrated that gut microbiota are closely related to respiratory health and disease, playing a vital role in the development of COPD, asthma, lung cancer and respiratory infections.^[12–14]^

Probiotics are living microorganisms that provide important health benefits to an individual when administered in sufficient quantities.^[15]^ Prebiotics are indigestible food components that may produce beneficial effects by selectively stimulating growth and/or activity of certain types of bacteria in the colon, thereby improving health of the individual.^[16]^ Synbiotics are usually composed of a mixture of live microorganisms and substrates that are selectively utilized by host microorganisms to confer health benefits.^[17]^

Emerging studies have shown that dietary supplementation with probiotics can reduce lung deterioration and hospitalization rates in patients with pulmonary inflammatory disease.^[18,19]^ However, the specific efficacy of probiotics, prebiotics, and synbiotics in the treatment of CB or COPD has not been clearly defined in the existing literature. This protocol describes a systematic review and meta-analysis designed to synthesize clinical evidence and evaluate the clinical efficacy of probiotics, prebiotics, and synbiotics in the treatment of CB or COPD.

## 2 Methods

### 2.1 Study registration

This study has been registered on OSF (https://osf.io/; registration number DOI 10.17605/OSF.IO/SP35M). This meta-analysis will follow the Preferred Reporting Items for Systematic Reviews and Meta-Analyses Guidelines for Protocols (PRISMA-P).^[20,21]^

### 2.2 Eligibility

#### 2.2.1 Types of studies

All randomized controlled trials (RCTs) from any country will be included without language limitation. Non-RCTs, repeated publications from the same trial, surgical intervention trials, animal trials, adverse drug reaction (ADR)-related studies, mechanism trials, cohort studies, case-control studies, and methodologically poor studies will be excluded.

#### 2.2.2 Types of participants

Patients who meet any accepted diagnostic criteria for CB will be included, such as the British Medical Research Council (cough and sputum for at least 3 consecutive months over 2 years) or the American Thoracic Society, the Global Initiative for Chronic Obstructive Lung Disease, the European Respiratory Society or the World Health Organization. There will be no restrictions on age, gender, and race/ethnicity.

#### 2.2.3 Types of interventions

Probiotics, prebiotics and synbiotics will be included regardless of dose, frequency of consumption, duration of treatment, route of administration, and administration regimen (either in combination or as a single preparation).

#### 2.2.4 Types of comparisons

Placebo, any effective treatment or non-drug treatment (regardless of dose, frequency, duration of treatment, route of administration, and administration regimen) will be included. Non-RCTs, repeated publications from the same trial, surgical intervention trials, animal trials, ADR-related studies, mechanism trials, cohort studies, case-control studies, and methodologically poor studies will be excluded.

#### 2.2.5 Types of outcome measures

##### 2.2.5.1 Primary outcomes

1. COPD exacerbation.
2. Changes in health-related quality of life.

##### 2.2.5.2 Secondary outcomes

1. Lung function decline, defined by a decrease in forced expiratory volume in one second (FEV1), forced vital capacity (FVC), or peak expiratory flow rate (PEFR).
2. Lower respiratory tract infection.
3. Adverse effects of treatment.
4. Hospitalization and mortality.

### 2.3 Search strategy

#### 2.3.1 Electronic searches

In order to ensure that all eligible studies in the electronic databases are identified, we will conduct an extensive literature search without restrictions on language or publication date. The Cochrane Library, EMBASE, MEDLINE, PubMed, Scopus, the Web of Science, the China National Knowledge Infrastructure, the Wanfang database, and the China Science and Technology Journal database (VIP Information Network) will be comprehensively searched from their inception dates until September 30, 2020. We will include all relevant RCTs to examine the efficacy of probiotics, prebiotics and synbiotics for CB or COPD. The search strategies to be used for all databases will follow the recommendations of the Cochrane Handbook for Systematic Reviews of Interventions (https://training.cochrane.org/cochrane-handbook-systematic-reviews-interventions) (Table 1). Search terms will be adjusted, if necessary, depending on the requirements of each database.

**Table 1:** Search strategies in PubMed.

Search	Search terms of query
#1	Chronic bronchitis [MeSH Terms]
#2	Chronic bronchitis [Title/Abstract]
#3	CB [Title/Abstract]
#4	Chronic obstructive pulmonary disease [MeSH Terms]
#5	Chronic obstructive pulmonary disease [Title/Abstract]
#6	Lung disease [MeSH Terms]
#7	Lung disease [Title/Abstract]
#8	Pulmonary Disease [MeSH Terms]
#9	Pulmonary Disease [Title/Abstract]
#10	Bronchitis, Chronic [MeSH Terms]
#11	(COPD OR COAD OR COBD) [Title/Abstract]
#12	#1 OR #2 OR #3 OR #4 OR #5 OR #6 OR #7 OR #8 OR #9 OR #10 OR #11
#13	(Probiotics OR probiotic OR prebiotics OR prebiotic OR synbiotics OR synbiotic OR exp Lactobacillus OR Lactobacillus OR lactobacilli OR exp Bifidobacterium OR exp Bacillus OR bacilli OR bacillu OR Clostridium butyricum OR clostridium butyricum OR streptococcus thermophill OR exp Escherichia coli OR propionibacteria) [Title/Abstract]
#14	Clinical trial [Title/Abstract]
#15	Randomised or randomized [Title/Abstract]
#16	Randomized controlled trial [Title/Abstract]
#17	Placebo [Title/Abstract]
#18	#14 OR #15 OR #16 OR #17
#19	#12 AND #13 AND #18

#### 2.3.2 Searching other resources

We will also retrieve unpublished data from ongoing studies in the NIH clinical registry Clinical Trials.Gov (https://www.clinicaltrials.gov/), the International Clinical Trials Registry Platform (https://www.who.int/ictrp/en/), the Australian New Zealand Clinical Trials Registry (https://www.anzctr.org.au/), and the Chinese Clinical Registry (http://www.chictr.org.cn/index.aspx). Other publications, such as relevant systematic reviews and meta-analyses, will be reviewed to identify additional trials.

### 2.4 Data collection and analysis

#### 2.4.1 Selection of studies

**Figure 1:**
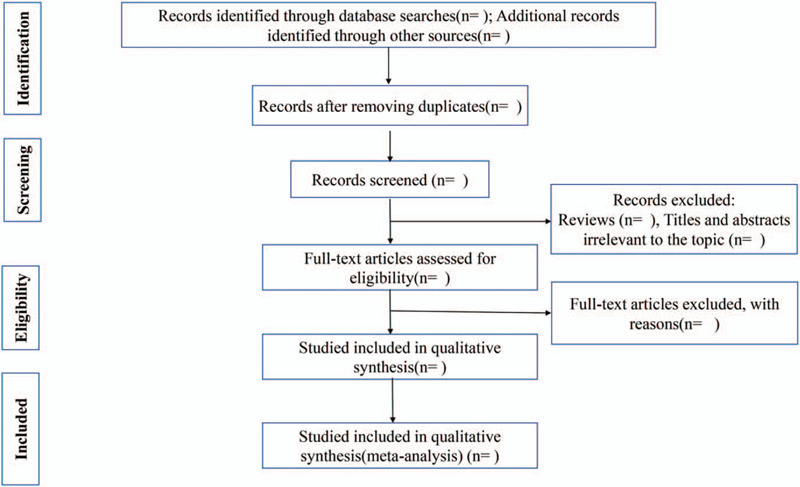
Flow diagram of the study selection process.

Two literature reviewers (WYC and PCX) will independently filter all publication titles and abstracts from each database search to identify potentially relevant studies and will then evaluate the full text of each study to determine if that study meets the inclusion and exclusion criteria. Any differences between reviewers will be resolved by discussion and consensus, and if necessary, will be adjudicated by a third author with clinical and methodological expertise. Details of the publication selection process are summarized in a PRISMA flow diagram (Fig. 1).

#### 2.4.2 Data extraction and management

The procedure for data extraction and management will be similar to the process described above for selection of studies. Two researchers (WYC and PCX) will independently extract data and attempt to reach a consensus by discussion. A third author (WF) will adjudicate any discrepancies in order to reach an agreement. We will request from the original author (s) any required data or details that were not reported in the original publication. The data will then be entered into Review Manager (RevMan) version 5.3 software (https://training.cochrane.org/online-learning/core-software-cochrane-reviews/revman for further analysis. For each included study, the following information will be extracted:

1. Basic information about the study, such as first author, year of publication, title, journal of publication including issue and page numbers, and digital object identifier if available.
2. Study design characteristics such as methods for randomization, blinding methods, study group allocation, country of origin, location, and setting.
3. Study parameters such as total sample size, subgroup sample sizes, and inclusion and exclusion criteria.
4. Characteristics of participants such as age, gender, ethnicity, disease diagnosis, disease duration, and baseline characteristics.
5. Specific information on treatment procedures, dosage and schedule of administration, and duration of treatment and follow-up in the intervention and control groups.
6. Outcome measures including pre-defined outcomes and other outcomes reported in the study.

#### 2.4.3 Assessment of bias risk

We will use the Cochrane Handbook for Systematic Reviews of Interventions to assess the quality of reviews. Results will be independently cross-checked by 2 reviewers, and following internal discussion, any remaining inconsistencies will be resolved by a third reviewer.

#### 2.4.4 Measures of treatment effect

Risk ratios (RRs) with 95% confidence intervals (CIs) will be used to evaluate dichotomous outcomes. Continuous data will be represented by the mean difference (MD) of the 95% CI, and if different measurement scales are used, by the standardized mean difference (SMD).

#### 2.4.5 Unit of analysis issues

Data from phase I randomized cross-over trials will be included in the meta-analysis. For studies involving multiple intervention groups, results reported in different units will be converted to the International System of Units before statistical analysis.

#### 2.4.6 Missing data

We will contact the corresponding author of any article that did not report necessary information in sufficient detail. If the needed information cannot be provided, the article will not be included in the analysis.

#### 2.4.7 Assessment of heterogeneity

We will use the *I*^2^ statistic to evaluate the percentage of variation across studies that is attributable to heterogeneity rather than chance.^[22]^ An *I*^2^ value ≥50% will indicate significant heterogeneity among studies. If the *I*^2^ value is <50%, a fixed-effects model will be used; however, if the *I*^2^ value is ≥50%, a random-effects model will be applied. If significant heterogeneity among studies is identified, we will conduct a subgroup analysis to examine possible causes, as described below. If the data allow, we plan to exclude selected trials that contribute the most to heterogeneity to determine their overall effect on the results.

#### 2.4.8 Assessment of reporting biases

If the meta-analysis includes more than 10 studies, funnel plots will be used to assess reporting biases.

### 2.5 Data synthesis

RevMan version 5.3 software (the Cochrane Collaboration) will be used for data synthesis. RRs with 95% CIs for dichotomous outcomes will be used to report effect size estimates. Continuous data will be presented as MDs with 95% CIs. The SMD statistic will be used to analyze continuous data if different measurement scales were reported. We will attempt to identify the causes of heterogeneity from various aspects and provide a narrative and qualitative summary.

#### 2.5.1 Sensitivity analysis

Sensitivity analysis will be used to confirm the robustness of the primary results, and to determine how methodological weaknesses, study types, missing data, sample size, and heterogeneity affect the meta-analysis results.

#### 2.5.2 Evaluation of the quality of evidence

The Grading of Recommendations Assessment, Development and Evaluation will be used to assess the quality of evidence.^[23]^ The assessment will be categorized using the following 4 levels:

1. Very low: very little to no confidence that the estimated result(s) reflect the true result(s), the true result(s) are likely to be substantially different from the estimated result(s).
2. Low: limited confidence that the estimated result(s) reflect the true result(s), the true result(s) may be substantially different from the estimated result(s).
3. Moderate: moderate confidence that the estimated result(s) reflect the true result(s).
4. High: high confidence that the estimated result(s) reflect the true result(s).

#### 2.5.3 Subgroup analysis

If the necessary data are available, subgroup analysis will be performed based on treatment time, blank controls, and method of delivery. We will explore the sources of any significant heterogeneity and will perform subgroup analysis based on those factors if they can be reliably identified.

## 3 Discussion

The benefits of probiotics, prebiotics and synbiotics have been widely used due to their confirmed effectiveness; however, their clinical efficacy and safety for treating CB or COPD are unclear. The primary goal of this systematic review is to methodically analyze the clinical efficacy and safety of probiotics, prebiotics, and synbiotics and to provide this information to clinicians and researchers to improve treatment of CB and COPD.

### 3.1 Amendments

Any amendments to this protocol will include the date of each amendment, a description of the procedural change(s), and the reason(s) that necessitated the change(s).

## Acknowledgments

We would like to thank TopEdit (www.topeditsci.com) for English language editing.

## Author contributions

**Conceptualization:** Caixia Pei, Liyun Liu, Yongcan Wu, Fei Wang

**Data curation:** Liyun Liu, Xiaomin Wang.

**Formal analysis:** Yongcan Wu, Caixia Pei.

**Funding acquisition:** Fei Wang.

**Investigation:** Caixia Pei, Liyun Liu, Xiaomin Wang.

**Methodology:** Caixia Pei, Liyun Liu, Yongcan Wu, Xiaomin Wang.

**Project administration:** Caixia Pei, Liyun Liu, Yongcan Wu, Fei Wang.

**Resources:** Fei Wang.

**Software:** Caixia Pei, Liyun Liu.

**Supervision:** Yongcan Wu, Fei Wang, Caixia Pei, Liyun Liu.

**Validation:** Liyun Liu, Caixia Pei, Xiaomin Wang.

**Writing – original draft:** Caixia Pei, Liyun Liu.

**Writing – review & editing:** Yongcan Wu, Liyun Liu, Fei Wang.
